# Graphene-Related Nanomaterials for Biomedical Applications

**DOI:** 10.3390/nano13061092

**Published:** 2023-03-17

**Authors:** Andreea-Isabela Lazăr, Kimia Aghasoleimani, Anna Semertsidou, Jahnavi Vyas, Alin-Lucian Roșca, Denisa Ficai, Anton Ficai

**Affiliations:** 1 Department of Science and Engineering of Oxide Materials and Nanomaterials, Faculty of Chemical Engineering and Biotechnologies, University Politehnica of Bucharest, Gh. Polizu St. 1–7, 011061 Bucharest, Romania; 2 National Centre for Micro- and Nanomaterials, University POLITEHNICA of Bucharest, Spl. Independentei 313, 060042 Bucharest, Romania; denisaficai@yahoo.ro; 3 National Centre for Food Safety, University Politehnica of Bucharest, Spl. Independentei 313, 060042 Bucharest, Romania; 4 University of Essex, Wivenhoe Park, Colchester, CO4 3SQ, UK; 5 Charles River Laboratories, Margate, Manston Road, Kent CT9 4LT, UK; 6 Drug Development Solution, Newmarket road, Ely, CB7 5WW, UK; 7 Department of Inorganic Chemistry, Physical Chemistry and Electrochemistry, Faculty of Chemical Engineering and Biotechnologies, University Politehnica of Bucharest, Gh. Polizu St. 1–7, 011061 Bucharest, Romania; 8 Academy of Romanian Scientists, Ilfov St. 3, 050045 Bucharest, Romania

**Keywords:** graphene-related (nano) material, bionanocomposite, stimuli-responsive drug-delivery system, biodegradability, tissue engineering, neuronal regeneration, biomedical applications, toxicity

## Abstract

This paper builds on the context and recent progress on the control, reproducibility, and limitations of using graphene and graphene-related materials (GRMs) in biomedical applications. The review describes the human hazard assessment of GRMs in in vitro and in vivo studies, highlights the composition–structure–activity relationships that cause toxicity for these substances, and identifies the key parameters that determine the activation of their biological effects. GRMs are designed to offer the advantage of facilitating unique biomedical applications that impact different techniques in medicine, especially in neuroscience. Due to the increasing utilization of GRMs, there is a need to comprehensively assess the potential impact of these materials on human health. Various outcomes associated with GRMs, including biocompatibility, biodegradability, beneficial effects on cell proliferation, differentiation rates, apoptosis, necrosis, autophagy, oxidative stress, physical destruction, DNA damage, and inflammatory responses, have led to an increasing interest in these regenerative nanostructured materials. Considering the existence of graphene-related nanomaterials with different physicochemical properties, the materials are expected to exhibit unique modes of interactions with biomolecules, cells, and tissues depending on their size, chemical composition, and hydrophil-to-hydrophobe ratio. Understanding such interactions is crucial from two perspectives, namely, from the perspectives of their toxicity and biological uses. The main aim of this study is to assess and tune the diverse properties that must be considered when planning biomedical applications. These properties include flexibility, transparency, surface chemistry (hydrophil–hydrophobe ratio), thermoelectrical conductibility, loading and release capacity, and biocompatibility.

## 1. Introduction

### 1.1. Tissue Engineering

The interdisciplinary field of tissue engineering (TE) comprises the use of biomaterials, biological active agents, including cells, and engineering to restore or develop biological substitutes for various tissues in the body [1]. Assembling these functional constructs may contribute to repairing damaged tissues or whole organs. As such, TE methods have emerged as useful techniques in tissue regeneration and organ transplantation. TE and regenerative medicine aim to replace or enhance damaged biological tissues by creating substitutes, preferably via a biomimetic approach. These fields of knowledge mix engineering, (micro)biology, and medicine [2]. Recent developments in these areas have generated significant interest in neural interfaces that can replace or enhance the function of the nervous system that has been impaired due to illness or injury. To help individuals with neurological disorders, neural interface technology aims to establish a connection between the external environment and nervous system by stimulating neural tissue [3]. 

### 1.2. Scaffolds in Tissue Engineering

Cells may be grown in two-dimensional (2D) or three-dimensional (3D) cultures. Generally, 2D cultures comprise the growth of cells on a flat surface, such as a flask or petri dish. This method is commonly used in research given its cost-effectiveness, feasibility, and ease of observation when analyzing cells; however, it lacks the capacity to accurately resemble in vivo cellular environments. On the other hand, 3D cultures, which may be performed using the hanging-drop method, microfluidic platforms, or scaffolds, allow for interactions between cells and the extracellular matrix (ECM) to take place [4]. As such, 3D cultures allow for improved cellular differentiation and proliferation compared to those in 2D cultures and mimic the morphologies and physiologies of in vivo cells and tissues in a more accurate manner.

Scaffolds are structures that are used in both in vitro and in vivo TE applications and serve as support to ensure the growth of the ECM, cell adhesion, and subsequent tissue growth [5]. Scaffolds are typically designed to resist external pressure while maintaining permeability to ensure the movement of nutrients and growth factors from the culture media used to grow the scaffolds to the cells. After scaffolds have fulfilled their role in tissue growth, the biomaterials used in their design and fabrication should degrade to allow the cells to continue proliferating independently.

The development of scaffolds that can encourage neural tissue regeneration has generated much interest for GRMs. Recent studies have demonstrated that GRMs encourage the adhesion, proliferation, and differentiation of various cells, including embryonic stem cells (ESCs), neural stem cells (NSCs), mesenchymal stem cells (MSCs), and induce pluripotent stem cells. Many researchers have found that scaffolds based on conductive materials can boost NSC proliferation and differentiation into neuronal or glial cell lineages [6]. GRMs are therefore promising nanoplatforms for treating neural tissue injuries in regenerative medicine.

In 2015, Jakus et al. demonstrated that human mesenchymal stem cell (hMSC) adhesion, viability, proliferation, and neurogenic differentiation may be supported by 3D printable graphene (3DG) [7]. These processes were found to occur in vitro in basic growing media without neurogenic stimuli. Glial and neuronal gene expression was also significantly upregulated. According to in vivo tests, 3DG exhibited promising biocompatibility for at least 30 days.

Molecularly changing the surfaces of neural probes with anti-inflammatory agents, adhesion of proteins, and bioactive chemicals comprises one method of regulating the inflammatory process and improving neural electrode incorporation into brain tissue [8]. Hyaluronic acid (HA), peptides, and growth factors are a few examples of biological surface modifications that may be used on neural devices. Other non-biological enhancements include hydrogels, conducting polymers [9], carbon nanotubes, and hydrogel coatings [10].

A significant challenge in using neural electrodes for recording brain activity both in vitro and in vivo is developing novel materials that produce seamless neural interfaces with high sensitivity. Another issue that has driven the creation of innovative materials is ensuring long-term stability. By considerably boosting the signal-to-noise ratio, electroactive nanomaterials such as silicon nanowires [11], carbon nanotubes [12], and conducting-polymer nanostructures [13] may be used to create durable and sensitive neural interfaces.

### 1.3. Properties of Effective Scaffolds

The ECM is a critical component of all types of tissues and serves as a natural scaffold for cells to adhere to and differentiate. The ECM, therefore, contributes greatly to the homeostasis and morphogenesis of tissues. In 3D TE, scaffolds may be developed using various polymeric or non-polymeric materials to mimic the ECM of target tissues. When selecting biomaterials for scaffolds, it is important to choose a material with properties that are suitable for the target tissue and cell types being used as the properties of the material should mimic those of the ECM of the target tissue. This will ensure that the material will contribute to obtaining the desired results.

The properties of various scaffolds can differ in terms of structure, rigidity, strength, pore size, and the concentrations of the polymers used [5]. The composition, cyto-, hemo- or tissue-compatibility, bioactivity, and mechanical properties of scaffolds should further be considered when selecting suitable biomaterials for the desired application. Natural materials, such as HA, cellulose, collagen, and chitosan, may be found in natural ECMs but tend to be highly degradable and difficult to purify. They also present the risk of inducing an immune response upon use [14]. Synthetic scaffolds, on the other hand, allow greater control and reproducibility as they can be synthesized in the manner that is desired. They can further be modified to circumvent immune detection. Hydrophilicity, hydrophobicity, and the surface charge of a scaffold are also properties that may determine the suitability of a scaffold for the desired use. Hydrophobic materials, for example, have been found to allow for cell adhesion but also present with a higher risk of inducing an immune response as they can allow for the increased adhesion of monocytes [14]. Other studies have found that hydrophilic materials are more effective than hydrophobic materials in terms of cell adhesion [15,16,17]. Positive surface charges have further been found to allow for increased cell adhesion compared to negative surface charges. This is because surface charges determine the level of protein absorption into the scaffold, which is correlated with cell adhesion [18]. Even if the exact mechanism is still not fully understood, the recognition of the GRMs by the immune system (including complement protein gC1q) is well correlated with the polarity and surface characteristics (which include the self-assembling capacity of the surface). 

## 2. Biomaterials for Tissue Regeneration

Biomaterial-related scaffolds provide porous networks, mechanical and structural support, shape, and hierarchical structures with hydrophilic properties to allow for cell attachment, cell–cell communication, proliferation, and differentiation for tissue regeneration in TE. To date, most synthetic biomaterials for TE applications may be generated from lactic acid, caprolactone, or glycolide monomers, which can form poly(L-lactide), polycaprolactone, and poly(L-glycolic acid) materials or copolymers [19].

Chitosan, alginate, starch, collagen, HA, cellulose, fibrin, silk, and their derivatives may also be utilized to build scaffolds [20]. To manage the lengths, thicknesses, porosities, and structures of biomaterial-related scaffolds for TE applications, a range of approaches and techniques have been explored [21]. Apart from collagen, other animal-derived proteins, such as laminin and Matrigel, have been used to create 3D scaffolds for various TE applications, including skin restoration, bone substitution, artificial artery construction [22], and cell transport. In order to create hydrogels for drug release and tissue regeneration, polysaccharides may be modified through crosslinking [23].

Recent studies have assessed the potential of silkworm silk as a unique biomaterial that may address many of the shortcomings of the current materials used in TE scaffolds. The natural protein, silk fibroin (SF), which is obtained from silkworm silk, has been studied as a potential biopolymer for TE due to its biocompatibility, tunable biodegradation, minimal immunogenicity, adaptability to different forms (such as 3D scaffolds, thin films, nanofibers, microspheres, nanoparticles, and hydrogels), excellent mechanical strength, and ease of accessibility [24,25]. SF can further be combined with other polymers to create composite scaffolds that support cellular differentiation, proliferation, and attachment [26]. SF-related biomaterials can also be utilized in various material forms, including solutions, powders, nanoparticles, fibers, mats, films, hydrogels, sponges, and 3D structures [27].

HA, a polymer that is composed of linear glycosaminoglycan and is abundant in the ECM, has been shown to impact cell signaling pathways, which is critical in TE. Clinical trials have further shown that HA is useful in treating osteoarthritis, and the US Food and Drug Administration has approved HA as a biomaterial for human use [28].

Stem cells are usually grown in vitro in a monolayer on a flat plastic substrate. This method, however, cannot fully replicate the characteristics of cells in their native microenvironments. Thus, it is important to offer an adequate microenvironment for developing functional tissues by creating biomimetic scaffolds that are analogous to stem cell niches [29]. Conductive scaffolds not only promote stem cell activities by electrical stimulation but can also offer microenvironments that include numerous biochemical and biophysical factors for optimum stem cell function [30]. To influence stem cell activity, several conductive materials, such as graphene, polypyrrole (PPy), poly(3,4-ethylene dioxythiophene), and polyaniline, have been utilized in scaffolds. These materials comprise repeated single and double bonds with conjugated π-bonds that allow free electrons to travel between atoms [31,32].

To date, various biological materials made of natural or synthetic materials have been used for peripheral nerve regeneration, including HA, SF, polypropylene alcohol, sodium alginate, gelatin, chitosan, and polyacrylamide (PAM) [33,34,35,36,37,38,39]. Although these biomaterials have excellent potential for use in treating peripheral nerve damage, their efficacy needs to be improved to the level that is required for the rapid regeneration and functional recovery of peripheral nerve damage in clinical settings [40].

Due to their excellent biocompatibility, ability to retain water, and drug-delivery capabilities, hydrogels have recently become widely used in TE including nerve regeneration [41]. For example, by chemically crosslinking a biodegradable HA hydrogel conduit, Ortuno-Lizaran et al. discovered that Schwann cells (SCs) can thrive in the conduit lumen [42]. In another study, collagen hydrogel-filled chitosan tubes containing ECM were found to be possible candidates for the treatment of nerve damage [43]. The conduit could stimulate the repair of peripheral nerve injury to some extent. Additionally, vascular endothelial growth factor-loaded hydrogels created by Xu et al. were found to enhance the regeneration of neurons [44]. Another study demonstrated that genipin crosslinked gelatin conduits can successfully encourage the regeneration of peripheral nerves [45].

Due to their excellent biocompatibility, PAM hydrogels are frequently used in the field of peripheral nerve regeneration [46]. Pure PAM hydrogels, however, often have poor mechanical properties and are brittle. Additionally, post-implantation hydrogel scaffolds may be distorted or even destroyed by other surrounding tissues, which renders them unsuitable for prolonged use in TE [47]. Therefore, improving the mechanical characteristics and biocompatibility of PAM hydrogels, such as by combining them with other materials, is necessary for the implementation of these hydrogel conduits. Huang et al. created a PAM/GO/gelatin composite hydrogel, which exhibited improved mechanical properties [48]. A PAM/GO/gelatin/sodium alginate composite hydrogel was also successfully prepared by Zhao et al. [49]. Sodium alginate with improved softness modified the mechanical characteristics of the hydrogel, and gelatin enhanced its biocompatibility and viscoelasticity. The composite hydrogel also successfully encouraged the formation of SCs.

## 3. Biomedical Applications of Graphene-Related Materials

One reason why graphene has drawn the interest of scientists worldwide is its advantage to be involved in a significant number of applications due to its unique characteristics [50]. It has been proven that 2D nanomaterial plays a key role not only in automotive, environmental, agricultural, and packaging application but also in biomedical practices [51,52,53,54]. Graphene is also involved in the carbomerization that leads to α-graphyne products [55]. Given the abovementioned properties, the use of graphene has facilitated a variety of applications in TE, cancer treatments, bioimaging, biosensing, DNA and RNA extractions, the production of medical devices, antibacterial, and antiviral materials for drug and gene delivery [56].

**Figure 1:**
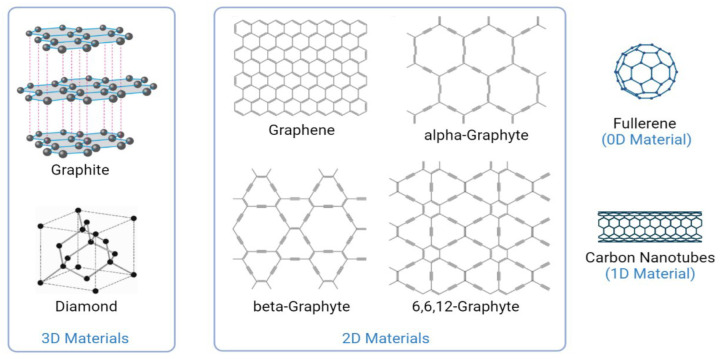
Chemical composition of graphene and processes in which it is involved. Graphene comprises a hexagonal carbon atom arrangement, which can form zero-, one-, and three-dimensional structures. Reprinted with permission from ref. [69]. Copyright 2012 American Physical Society.

Graphene is an especially intriguing material as it is composed of carbon in sp^2^ hybridization, which makes it electroconductive (Figure 1). Carbon materials are known for their mechanical strength, stability, and biocompatibility [57]. Biocompatibility is of the utmost importance when selecting materials for scaffolds as it allows for scaffolds to be used safely without eliciting toxicity or an immune response [58]. Graphene is derived from graphite and is an allotrope of carbon. These two materials have very different properties even if they have the same composition. Graphene consists of a monolayer of carbon atoms that comprises a honeycomb-shaped lattice [59]. The atomic structure of graphene allows for its thinness, tensile strength, large surface area, stability, and elasticity, which have made it a material of interest in studies developing drug-delivery platforms, bioimaging techniques, bioelectronics, and TE methods [60]. Even if graphene has a similar hexagonal structure as does graphite, the 2D structuration is leading to a special structure where all the C atoms are on the surface (if we are talking about the theoretical graphene with only one layer) and consequently the (surface) reactivity is highly increased. 

Careful examinations of graphene-related materials by scientists have provided insights into its composition, nature, and properties. Its high surface area, excellent electrical and thermal conductivity, high transparency, mechanical strength, increased elasticity, flexibility, resistance, and tuneable hydrophil-to-hydrophobe ratio have proven its great potential and promising use in different biomedical applications [61,62,63,64,65,66,67,68].

### 3.1. Graphene-Related Materials in Biosensing and Bioimaging

Apart from the exploitation of the properties of graphene in TE, the properties have also been found to be useful for biomedical applications such as biosensing and bioimaging. Optical imaging, emission tomography, magnetic resonance, photoacoustic, Raman spectroscopy, and multimodal imaging are a few of the methods that graphene has been associated with [70]. Furthermore, graphene and its derivatives have been used to promote the development of several distinct biosensors due to their high surface area, ability to conduct electricity, flexibility, and capacity to quench fluorescence [71,72]. These properties are not only useful in the development of enhanced disease-inducible microorganisms and marker monitoring to inspect blood, urine, sweat, and saliva but are also useful in drug discovery [73]. 

Photoluminescent materials are known to have significant optical properties [74]. When combined with GO, a significant enhancement in these optical properties may be observed due to the internal structural gaps of the nanomaterial. The addition of numerous oxygen-comprising functional groups into GO and quantum dots (GOQDs) [75], which are particles that comprise optical [76] and electrical qualities [77], has been found to play a key role in the photoluminescence of large wavelengths by modulating their band gaps. The photoluminescence of GOQDs has also exhibited quenching under distinct conditions and may therefore facilitate medical imaging applications and biosensor production [75]. Graphene and its derivatives have also been used in other techniques, such as laser desorption and ionization of mass spectrometry (LDI-MS) for screening purposes [78]. In LDI-MS, molecules are ionized and analyzed following the use of high-voltage power. In particular, the use of GO paper in LDI-MS analyses has revealed biogenic amines in their primitive stages [79]. Biogenic amines are solutes that regulate biological functions in the body [80]. Generally, the LDI-MS technique decreases the amount of time needed for complex sample procedures. It also simultaneously enables rapid screening [78] for the recognition of metabolites, peptides, proteins, bacteria, and other medical compounds to create drugs [81]. The technique has also been used to control and standardize the previously discussed biosensors [82]. 

Graphene has also been used in DNA and RNA analyses. Fani et al. [83] illustrated that electrochemical DNA biosensor analyses and T-lymphotropic virus-1 (HTLV-1) detection progressed successfully upon the use of differential pulse voltammetry (DPV). The utilization of single-stranded DNA probes, PPy, and rGO-PPy-AuNPs together with GOs were important for this process. Béraud et al. [84] further discussed the use of graphene in graphene field-effect transistors, which are biosensors with high electrical conductivity that carry out small molecule, ion, protein, and DNA analyses. 

For example, DNA and RNA nucleotide base structures have been observed through hexagonal boron nitride heterostructures and graphene. Single electron transistor devices have also been found to be valuable for detecting nucleotide bases and sensitivity. Boron nitride and graphene have been found to exhibit high levels of strength, which aid the binding activity of the bases [85]. Li et al. [86] further unveiled the patterns of RNA in the presence of graphene and found that the patterns and behavior of RNA were affected by the number of layers, concentration, and temperature of graphene.

### 3.2. Graphene-Related Scaffolds

Nanomaterials have been used widely for the generation of TE scaffolds, which may be used for cell differentiation, growth, and tissue regeneration [87] but could also represent concerns related to their degradation and clearance [88]. Blood vessels, cartilage, muscles, bones, and an extensive range of organs [60] are a few examples of areas where GRMs enable the growth and modulation of tissues by activating proteins, enzymes, and growth factors and thus have a direct impact on cell viability, adhesion, and growth, as proved by Lee et al. [89]. Another study showed that the combination of soft polyoxyethylene sorbitan laurate and graphene facilitated the successful adhesion of African green monkey kidney cells, bovine embryonic cells, and Crandell–Ress feline kidney cells and highlighted the biocompatibility of the material [90].

Graphene-related scaffolds should be designed in a way that is compatible with their intended use and the cells that will be cultured. They should further be biodegradable/ resorbable and release byproducts that are not toxic to the host. It is also important that they are porous so that they will allow for the adhesion and growth of cells and the transport of metabolic waste [91]. There are various conventional and rapid prototyping methods that may be used to fabricate graphene-related scaffolds. Conventional methods include solvent casting, electrospinning, gas foaming, phase separation, lyophilization, and leaching [92,93,94]. These methods, however, do not allow for optimal control over the parameters of the scaffolds, such as the sizes of the pores and overall structures of the scaffolds. This makes it difficult to reproduce scaffolds consistently [91]. Rapid prototyping methods, which include 3D printing techniques such as stereolithography and selective laser sintering, use computer-aided design software to circumvent these issues [5,94,95]. Such methods allow for greater accuracy when producing scaffolds as they allow for scaffolds to be fabricated in a layer-by-layer approach to precisely match the parameters of the target tissue [96].

The properties of GRMs allow them to be an effective material for building a wide range of scaffolds, as in most of them the mechanical properties especially are exploited. Osteoblast cells have been assessed in studies on graphene nanoparticles to determine the suitability of the nanoparticles for use in bone treatment scaffolds and bone regeneration, especially because it can significantly enhance the mechanical properties of these grafts. Kalbacova et al. [97] tested the biocompatibility of graphene by assessing whether SAO-2 human osteoblast cells and MSCs would adhere to it. This experiment verified that osteoblast expansion and development occurred at a level that was twice as significant as the expansion and development of the osteoblasts when tested with SiO_2_. More significant proliferation and differentiation, therefore, occurred in the presence of graphene. This further indicates that graphene may be a valuable molecule that plays a crucial role in bone tissue regeneration [98].

In skin tissue engineering, GRMs were also tested and found as a viable alternative especially in association with several biopolymers, including collagen and gelatin, chitosan, polycaprolactone, etc. For instance, complex polycaprolactone-graphene oxide-silver-arginine quaternary systems were used trying to combine the beneficial activity of the components, including the antimicrobial activity of the silver nanoparticles, while GO-Arginine was responsible with the upregulation of endothelial nitric oxide synthase (eNOS) expression [99]. Graphene-oxide modified electrospun polyvinyl alcohol nanofibrous scaffolds were also obtained and proved to be efficient wound dressing, the composition with 0.25% GO proving an over 90% contraction of the wound within 9 days [100]. 

TE has allowed for improved diagnostic and therapeutic techniques. Tissue and organ transplantation methods may also be improved through TE with various biomaterials. Biomaterials have been proven to be useful in developing biomimetic tissue constructs; however, different host tissues have different physical, chemical, and electrical properties, which individual biomaterials may not be able to replicate independently. Developing composite or hybrid materials is therefore necessary to ensure each property of a tissue is mimicked effectively [95]. Graphene-related scaffolds have also been found to allow for the efficient differentiation of stem cells from various cell lines, which has been useful in studies on therapies for damaged livers, brain, heart, etc. [4]. 

Lu et al. demonstrated that GO can interact with DNA oligonucleotides via multiple interactions and thus could be used to protect them from enzymatic cleavage while delivering the oligonucleotides into target cells [101]. Tang et al. found similar results and further showed that graphene sheets could adsorb oligonucleotides onto their surfaces to enhance their specificity to complementary DNA strands [102]. These features are important in gene therapy techniques, which require the protection of DNA from cleavage and efficacy of the delivery of genetic material into target cells. Given that other studies have indicated the potential use of both viral and nonviral vectors in delivering genetic material to target cells, GRMs may potentially be used as non-viral vectors [103,104]. This could especially be true given their nanoscale sizes and resultant ease of uptake when entering cells [105,106]. Other studies have assessed the combined use of graphene and its derivatives with other biomaterials to amplify their physical, mechanical, and electrical properties. These properties, as well as the surface properties of the biomaterials and their resulting scaffolds, can influence the adhesion, differentiation, and proliferation of cells while the delivery can be triggered even by electric fields. Poly(Lactic Acid)/Graphene Oxide/Quercetin Fibrous Scaffolds can be used as an electrical triggering system for wound dressing. Depending on the characterististics of the electric field, the delivery can be enhanced over 8000 times if an electric field characterized by 10 Hz is used [107]. Moreover, it has been found that coating graphene onto a silicon dioxide surface can enhance the adhesion and proliferation of hMSCs and osteoblasts better than silicon dioxide can alone [97]. Other studies have shown that combining graphene with polyelectrolytic materials, such as poly-l-lysine, can control the electrical resistance of the materials and subsequently facilitate the adhesion of neural cells and outgrowth of neurites [108].

**Figure 2:**
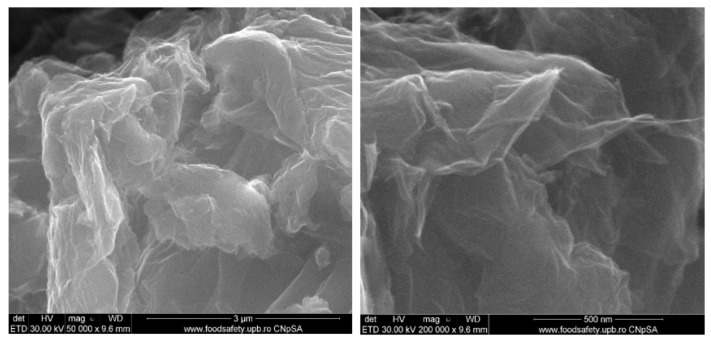
Microscopy analysis of a graphene oxide support morphology at 1000×, 10 kX. Reprinted from [116].

Various methods may be used to characterize 3D graphene-related scaffolds. For example, the morphology of a graphene-related scaffold may be determined using transmission electron microscopy or atomic force microscopy imaging [109,110]. Raman spectroscopy may be used to assess the chemical structure of the scaffold [97]. The surface chemistry of the scaffold may be analyzed using X-ray photoelectron spectroscopy (XPS), which would allow for the elemental and atomic compositions of the scaffold to be assessed [111,112]. In graphene-related scaffolds, XPS can determine changes in the carbon-to-oxygen ratio, which can further indicate the reduction in GO [4]. The mechanical properties of graphene-related scaffolds may be characterized by measuring the tensile strengths of the scaffolds, their load-bearing capacities, and the elastic, flexural, and Young’s moduli [113,114]. Various assays may further be performed to assess the biocompatibility of a scaffold. These include cell viability, staining, lactate dehydrogenase, and reactive oxygen species (ROS) assays [97,115]. Figure 2 highlight two representative SEM images of the graphene oxide at two various magnifications [116].

### 3.3. Graphene-Related Scaffolds in Neural TISSUE engineering

Graphene has the capacity to simulate the complex signaling systems in the nervous system—neurons connect electrical messages using chemical signals, such as acetylcholine [117]—given its conductivity and biocompatibility [118]. 

GRMs have been utilized in brain interfaces and electrical recordings due to their electrical properties [119,120]. A unique technique for enhancing the recording of neuronal data or triggering neuronal activity has emerged and involves the development of an expanded neural interface that combines graphene and the neurobiology [121]. A recent study evaluated the effects of graphene electrodes on the neural activity of differentiated neurons [122]. A network formed of graphene sheets demonstrated an increase of approximately 30% in neuronal signaling after the formation of the neural network. Spontaneous Ca^2+^ oscillations in the neurons doubled in comparison with that in the control group. These findings suggest that GRMs may encourage the growth of neural networks and improved brain circuits. Liu et al. developed an implanted graphene-related brain electrode to track electrophysiological and neurochemical signaling in vivo [123]. To measure the concentration of hydrogen peroxide in an in vivo hyperacute stroke model, an electrode wrapped in a rGO/Au_2_O_3_ nanocomposite was developed. This rGO-modified electrode provided increased electron transport between tissue and electrode surfaces, lower detection limits, and greater sensitivity to hydrogen peroxide than standard gold electrodes did. Feng et al. also exhibited the use of soft nanofibers based on graphene for the monitoring of brain activity [124]. Negatively charged graphene nanofibers (G-NFs) were produced on poly (vinyl chloride) nanofibers that had undergone NH_3_ plasma treatment prior to the reduction in graphene oxide nanofibers (GO-NFs) to produce the G-NFs. The ultrathin graphene shells of the G-NFs, which completely enveloped the nanofiber surfaces, resulted in high electrical conductivity and excellent flexibility. Thus, neurons on the G-NFs generated more neurite branches and expanded faster than neurons on the graphene films and tissue culture plates did. Neurons on the G-NFs also exhibited elevated levels of class III beta tubulin (TUJ1) and microtubule-associated protein 2 (MAP2) due to the geometric cues of the G-NFs, which closely mirrored nanofibers and mature neuronal markers. By applying electrical stimulation on neurons through G-NFs (referred to as G-NFs/ES), a growth rate that was noticeably boosted and approximately twice as fast as that of neurons on the G-NFs was identified. The activation of the calmodulin-kinase pathways by Ca^2+^ influx, which is driven by the depolarizing effect of the voltage-gated Ca^2+^ channels induced by G-NFs and increases intracellular Ca^2+^ concentrations, may have been the reason for this stimulation. Furthermore, the 3D G-NF nanofibers had a considerable impact on the development and growth of motor neurons. The G-NFs provided an anisotropic electron transfer at the points where the neurons made contact, which could have led to localized electrical stimulation.

Numerous studies have highlighted the use of graphene-related scaffolds in facilitating the adhesion and differentiation of neural cells [125,126]. Nerve cells are electrically excitable and transmit electrical signals via transmitters and synapses. They cannot undergo mitosis, however, and therefore cannot regenerate themselves [127,128]. It is therefore important to promote the differentiation of stem cells into neurons and electrical stimuli can be used in this regard [129,130,131]. The conductivity of graphene has further been demonstrated to stimulate neural growth [132,133]. For example, Aznar-Cervantes et al. found that electrospun SF scaffolds coated with rGO became conductive and improved the adhesion of PC-12 cells better than SF scaffolds did alone. When combined with electrical stimuli, the SF-rGO scaffolds allowed for the differentiation of the PC-12 cells into neural cells [134]. Zhang et al. [135] further found that coating GO onto poly-L-lactide nanoscaffolds could promote the neuronal differentiation of PC-12 cells. Other studies have shown that graphene and its derivatives may also be used as carriers to transfer growth factors from culture media to target cells, which further supplements the growth of the desired cells [133,136]. Overall, these findings indicate that graphene is a highly conductive material and can facilitate the differentiation of neural cells. Graphene-related scaffolds may therefore potentially advance TE and therapeutic strategies involving nerve cell differentiation and regeneration to ultimately improve treatment options for patients with diseases of the nervous system [60,119,137].

The “gold standard” for healing damaged nerves or gaps in clinical settings is autologous nerve grafting with sutures. Long defects or gaps are challenging to fix due to the slow rate of axon regeneration (approximately 2–5 mm/day). A sufficient supporting bridge, also known as a conduit, is therefore needed to reconnect damaged nerves [138].

In a study by Hong et al. [139], a 2D graphene layer produced on catalytic copper by a chemical vapor deposition method not only demonstrated excellent biocompatibility but also effectively simulated neurite outgrowth, suggesting that the graphene-related material was a viable option for treating injured nerves. Several studies have also shown that external electrical fields can promote neurite outgrowth and affect the directional control of neurons [140,141].

Li et al. [109] further cultivated NSCs on 3D graphene foams (3D-GFs) and observed enhanced neuronal growth. In addition to promoting NSC proliferation, the GFs had large specific surface areas (200–800 m^2^/g) and 3D porous structures. Additionally, their interconnected gaps improved cellular communication and allowed for efficient nutritional mass transfer for cells. Furthermore, the 3D-GFs displayed microscale topographic features, such as curvatures or anisotropic microstructures, which was in contrast with the features of 2D GRMs. These findings indicate that GFs may facilitate enhanced neuronal differentiation.

Despite the fact that GRMs have been extensively used to create films [123] or 3D scaffolds [109,124], there are ongoing attempts to expand the adaptability and applicability of graphene and its chemical derivatives for neurological regenerative medicine. It may be feasible to build complex neurological structures, such as the brain or neural conduits, using 3D printing technology.

**Figure 3:**
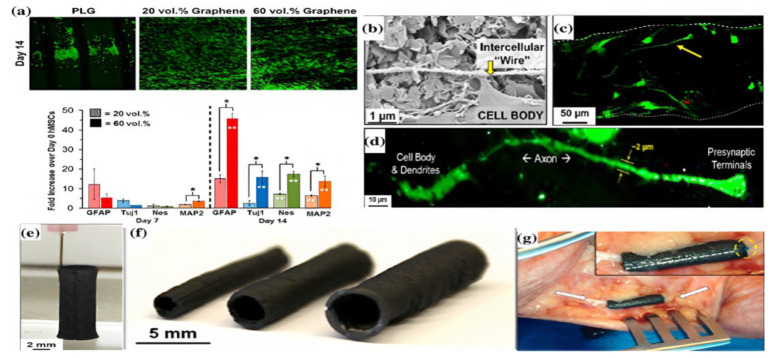
(**a**–**d**) 3D confocal reconstruction of human mesenchymal stem cells that were implanted onto different scaffolds for 14 days and marked as living (green) or dead (red). For 1, 7, and 14 days, the cells were planted onto scaffolds with 20% and 60% concentrations of graphene. The expression of the neurogenically relevant genes was altered to match that on day 0 (unseeded cells). (**e**,**f**) The 140-layer tubular structures were made using 3DG printing. (**g**) 3DG nerve conduit in a human cadaver. Reprinted with permission from ref. [7]. Copyright 2015 American Chemical Society.

Jakus et al. used graphene and poly(lactide-co-glycolide) (PLGA) to generate a bioink, which they then utilized to 3D print a nerve conduit with a specific size [7]. They demonstrated that when graphene concentrations were raised from 20% to 60%, strain ranges could be reduced from 210% to 81%, and conductivity could increase from 200 to 600 S/m. After 14 days of growth on 20% and 60% 3D-printed graphene (3DG), strong proliferative behavior was observed in hMSCs cultured on the 3DG. Moreover, the expression of the neuronal-specific markers TUJ1 and MAP2 significantly increased. It was also shown that 3DG had an effect on hMSCs that resulted in neuronal induction in a basic growth medium free of neurogenic triggers (Figure 3a). On the 60% 3DG scaffolds, a high aspect ratio expansion of neurons (greater than 100 m) was also seen. The development of a network of wires connecting particular cells by cells on the 3DG scaffolds was noted (Figure 3b–d). A close examination of the cells identified the presence of presynaptic terminals and 2-μm-wide axon-like processes, which are characteristics of uni- or multi-polar neuronal morphologies (Figure 3d). Additionally, the 3DG conduit was coiled around the nerve bundle, connected along the nerve conduit, and then linked to the epicedium and nerve bundle by sutures after being used on a human cadaver model (Figure 3e,f). These findings indicate that the robust mechanical properties of the graphene-related conduit may be advantageous during surgical procedures (Figure 3g) [142].

In a study by Feng et al. on the influence of GO and rGO membranes on the differentiation of adipose stem cells (ADSCs) into nerve cells, it was discovered that GO had a stronger effect on the ability of ADSCs to differentiate into neurons after 7 days of culture [143]. One of the most frequently utilized cell lines in neuroscientific research, including investigations into neurotoxicity, neuroprotection, neurosecretion, neuroinflammation, and synaptogenesis, is the PC-12 cell line [144]. In a study by Corr et al., PC-12 neuronal cells were found to proliferate quicker on a graphene/chitosan membrane than on a graphene/poly(D, L-lactic acid) membrane [145].

Another possible treatment option for neurodegenerative illnesses involves the growth of NSCs, according to Huang et al., who embedded NSCs in hydrogels that were made by combining graphene or GO with polyurethane (PU) using 3D bioprinting [146]. The viscoelastic qualities of the hydrogel enhanced cell survival and oxygen metabolism, which increased 2–4-fold while containing only a minimal amount (25 ppm) of graphene nanoparticles. Additionally, although NSC expression is not visible in PU, the NSCs implanted in the graphene/PU or GO/PU hydrogels exhibited marked tubulin and glial fibrous acidic protein expression, which allowed for NSC growth.

In a study by Niu et al. [147], rat induced pluripotent stem cells (IPSCs) were cultivated in a thin film made of SF and graphene. The graphene/SF membrane was found to support the neural differentiation process of the IPSCs as graphene can carry electrical stimuli and drive neuronal growth. The degree of neural differentiation was further found to increase as the concentration of graphene increased.

In another study by Magaz et al. [148], electroactive SF/rGO nanofiber scaffolds were created by adding a 10 wt% concentration of GO to SF and performing in situ reductions. The surface roughness and protein adsorption capacity of the scaffold were found to increase as the volume fraction increased. The reduction treatment significantly improved the conductivity of the scaffold compared to that of the SF/GO samples and increased the proliferation of neuronal NG108-15 cells, which encouraged protrusion and expansion.

In order to create a GO/antheraea pernyi silk fibroin (ApF)/poly(L-lactic acid-co-caprolactone) (PLCL) scaffold, Wang et al. coated GO onto an electrospun ApF/PLCL composite scaffold [149]. Following the addition of GO, the mechanical properties of the ApF/PLCL scaffold and the hydrophilicity of the stent were improved. The GO-coated ApF/PLCL scaffold also greatly increased PC-12 cell differentiation, SC migration, proliferation, and myelination, and upregulated the expression of focal adhesion kinase. This scaffold effectively healed a 10-mm sciatic nerve lesion in vivo and demonstrated a healing capacity comparable to that which follows autologous transplantation [150].

### 3.4. GRMs for Teranostics

Since graphene is a multifunctional carbon nanostructure, it is not infrequent for it to be utilized in cancer treatments and therapies. QDs, other carbon nanomaterials, GO, and graphene itself are usually involved in processes such as tumor screening, chemotherapies, and photothermal and photodynamic therapies [151]. Graphene can also be considered a nanocarrier for anticancer drugs in cancer cells [152].

The surface of graphene has been found to exhibit covalent and noncovalent activities, which are of utmost importance for its interactions with cancer cells, tissues, and anticancer drugs [153]. Various studies have described graphene as a “smart” nanomaterial due to its ability to activate its therapeutic qualities by taking advantage of tumor microenvironments and pH as well as the endogenous and exogenous stimuli [154]. 

Acidic pH and hydrogen peroxide are two of the features that play a vital role in the activities of tumor microenvironments. Lin et al. [155] showed that a specific type of graphene oxide (N-GO) can mimic hydrogen peroxide-related activities. Such activities include elevating the levels of ROS and turning hydrogen peroxide into ROS-hydroxyl radicals (HO) in Hela tumors, which results in the necrosis of the cancer cells. Although HO radicals show high toxicity levels, they do not negatively affect normal cells and tissues. Overall, these effects can be advantageous since drug resistance to tumors can be decreased, side effects on normal cells and tissues can be diminished, and significant biocompatibility can be achieved for a number of tumor therapies. 

GRMs are attractive for drug delivery and gene delivery especially because their surface can be tuned easily from highly hydrophobic to highly hydrophilic (according to the numbers of graphene sheets as well as the physically or chemically conjugation with different molecules such as, for instance, polyethylene glycol, surfactants, etc.). A proper functionalization can lead to a proper hydrophyl-hydrophobe ratio and thus the delivery rate can be adapted according to the needs. Moreover, GRMs are able to absorb visible and infrared light and thus photothermia can be generated with the main aim to act as a triggering factor in delivery. These findings highlight graphene as an ideal platform for gene and drug-delivery technologies [156]. 

Gene therapy is a medical approach that either treats or prevents disease by targeting genetically and adversely altered genes through various nanocarriers, such as graphene [157]. Lo et al. [158] found that graphene quantum dots (GQDs) could be used to eliminate colon cancer tumors in mice. Polyethylenimine and green fluorescent proteins (GFPs) were used to covalently bind the GQDs to the cancer cells. In this way, tumor cell membrane permeability was achieved, which was then followed by doxorubicin administration. In short, the GQDs provided a higher level of inhibition in the colon cancer tumors when combined with doxorubicin as opposed to using the drug alone. Lower toxicity levels were also found in the presence of the GQDs alone. 

GO has also been used as a nanocarrier for methotrexate, a drug against cancer cells, to test if the toxicity of the drug can be enhanced in tumors. Abdelhamid and Hussein [159] tested the drug in combination with graphene with hepatocellular carcinoma cells (HepG2), human embryonic kidney cells (HEK293A), and porcine skin fibroblasts (PEFs). The results revealed high toxicity levels in the cancer cells (HEK293A) in contrast to the normal cells (HEK293A and PEFs). Other studies have shown that drugs naturally tend to migrate toward nanocarriers. Various drugs have also been found to form π–π interactions and hydrogen bonds with the surface of graphene [160]. 

### 3.5. Graphene-Related Materials for the Treatment of Infections

It is also critical to consider the antimicrobial and antibacterial activities of graphene to achieve an in-depth understanding of its mechanisms and functions for biomedical applications. Biofilm formation is one of the most common ways to study a microorganism colony [161]. However, ensuring that biofilms and other materials are not contaminated by microbes is challenging. Antimicrobial resistance further threatens the effective prevention and treatment of diseases. It is therefore necessary to discover novel antimicrobial agents that will act against antimicrobial (multi) resistant bacterial strains resistance. Such agents may include graphene-related materials [162].

Various microbes have been identified for use in medical devices such as ventilators, urinary catheters, and dentistry devices. Developing devices with antimicrobial coatings is therefore necessary to diminish contamination levels. GRMs can be utilized to create suitable nanomaterial layers or device coatings to eliminate microorganisms [163]. Graphene and GO-coated aluminum plates have been used in studies on the microbial activity of *E. coli*. The results revealed the activity of *E. coli* was restrained and high levels of antimicrobial efficacy were achieved on the graphene and GO aluminum plates. Additionally, graphene nanowalls have been used as coatings for stainless steel-related materials and exhibited high activity against Gram-positive and -negative bacteria. The bacterial membranes appeared to be damaged following their exposure to the sharp edges of the graphene nanowalls [164]. 

**Figure 4:**
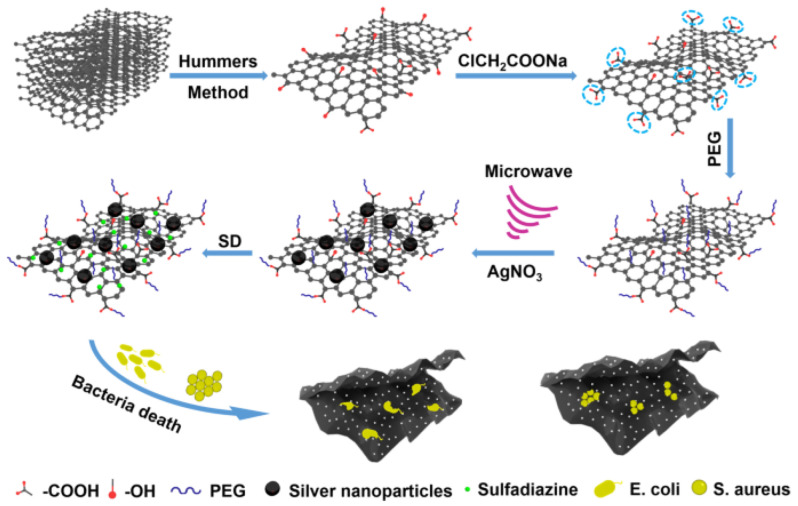
Preparation of the hybrid antibacterial system. Adapted from ref. [166].

Along with the intrinsic antimicrobial activity of the GRMs, more complex formulations based on GRMs and metal and metal-oxide nanoparticles, polyelectrolytes or antimicrobial agents were proposed. Certainly, the GRMs/Ag(/Polymer) or GRMs/ZnO(/Polymer) binary(/ternary) systems are some of the mostly studied GRM-based antimicrobial supports [165]. A special attention is paid to the development of drug-delivery systems based on GRMs and antimicrobial agents. Figure 4 highlights the preparation of a complex, hybrid antimicrobial system based on PEGylated GO and silver nanoparticles and loaded with antimicrobial agents such as sulfadiazine [166].

Some of the most important applications of the GRMs are presented in Table 1. 

### 3.6. Other Property-Derived Applications for GRMs

It has further been found that the high surface area of graphene in conjunction with its high chemical stability [167] enhances the catalysis of chemical reactions [168], which is of utmost importance in the production of electrochemical devices for biomedical applications. Zhou et al. [169] established that the activation of potassium hydroxide electrolytes can lead to greater graphene catalytic activity, which can be exploited for the neural electrode technique. This tool detects and regulates neural activity. According to Wei and Wang [170], examining the transmission of information between the nervous system and medical devices is fundamental in neuroscience since observations on neural activity can aid in the discovery of potent medicines and cures for neurological diseases. For the development of successful medical devices, neural electrodes, which can also measure neural activity when combined with graphene, must show excellent biocompatibility, minimal resistance, cause minimal damage, and balance neural activity and regulation [171,172,173]. Graphene may therefore be used in neuroscience and related biomedical applications as it exhibits all of the above characteristics. 

NeuroMem, a neurodevice that exploits graphene to mirror the activity of synapses in the brain, is an extraordinary and promising development for future practices in the field. It is a low-cost memristor device that not only portrays behavioral and neurological data but also provides access to memories through enhanced neuroimaging [174]. Graphene generally holds exceptional electrical properties and is usually employed as a conducting channel, charge-storing layer, or transparent electrode [175]. It is therefore widely used in memristor devices due to its high conductivity levels. NeuroMem combined with a reduced GO-synthesized (prGO) film has been found to be non-volatile, with the resistance of the device being manageable by adjusting the device value within specific ranges. This memristor is therefore a promising discovery in neurology and artificial intelligence [174]. 

## 4. Toxicity of Graphene-Related Materials

Graphene has been given the title “material of the future” and is increasingly being introduced in all the fields within science. However, it is necessary to consider the potential toxic effects of graphene in animal and human health. Over the years, environmental and biological safety concerns have been raised about the use and risks of graphene. Although graphene appears advantageous due to its abovementioned properties, studies have shown that it is associated with cellular apoptosis, necrosis, autophagy, cytoskeletal disorders, organelle and protein dysfunction, oxidative stress, physical destruction, DNA lesions, and inflammatory responses [176,177]. 

Graphene can also appear noxious if it is conglomerated in inordinate amounts in tissues and organs [176], such as the lungs [178], since it can lead to cellular damage and functional impairments. Generally, the toxic activity of GRMs is attributed to their lateral sizes, surface structures, functionality, and torque to consume proteins [176]. The most common way for individuals to be at risk following exposure to graphene is through its inhalation when in the form of dry powder [179]. 

The type and quality of graphene are significant factors that must be considered in biomedical applications since they can affect mammalian cells and lead to cell membrane lesions and, consequently, apoptosis. These issues comprise some of the major challenges in the commercialization of graphene. There is therefore a need to develop GRMs with exceptional quality and in large amounts with little cost [64]. 

Despite the abovementioned challenges, the focal point in developing GRMs is controlling the size of graphene products, which will allow for toxicity to be diminished in animals and humans [89]. In a study by Yang et al. [180], polyethylene glycol (PEG)-functionalized graphene did not deteriorate the liver and kidney functions in mice for 3 months. Most recent studies have suggested that using GRMs that can be easily excreted from and degraded in the human body can also aid in controlling toxicity. According to toxicologists, toxicity control can also be achieved by modifying the chemical composition of graphene or by administering it discontinuously. Experts have also reported that no risks have been recorded in the industrial applications of graphene [181]. 

The toxicity of graphene can also be reduced by forming polymer nanocomposite molecules with GRMs. The materials can be constrained through the polymer matrix nanocomposites, which further restricts their permeability. Enriched oxygen graphene molecules can further diminish the hydrophobicity of polymeric matrices to assist in cell adhesion and expansion [64]. 

By elucidating the mechanisms of graphene and considering the current comprehension of its functionalities, it can be assumed that not all GRMs can necessarily be characterized as hazardous. Likewise, not all of the materials can be characterized as harmless [177]. Numerous studies have reported that graphene is not toxic, especially in low amounts, and that graphene can be produced safely for use in biomedical applications. Moreover, graphene is made from carbon, which is a generally harmless material. Nevertheless, the potential toxicity and risks of using graphene in biomedical applications require further assessment, especially since several studies have highlighted that graphene can become hazardous at certain stages of the development of products [181]. 

Despite there being many studies that demonstrate the promising characteristics of graphene in scaffolds for nerve TE, implants, and therapeutic drug-delivery vehicles, several studies have shown that graphene can exert toxicity in the host or to cells [66,182,183]. The extent of its potential toxicity, however, remains unclear. This is due to several reasons. For example, graphene sheets vary in size and have several derivatives, which comprise different physicochemical properties and may therefore exert variable levels of toxicity [184]. Moreover, numerous studies have found that graphene can exert toxicity in a dose-dependent manner both in vitro and in vivo [185,186,187]. For example, Wang et al. [185] found that GO could cause cytotoxicity in human lung fibroblasts and decrease cell adhesion after 24 h when administered in doses greater than 20 μg/mL. Zhang et al. [187] further found that graphene itself could cause cytotoxicity in human neuronal cells when administered in doses of 10 μg/mL. Other studies have noted different thresholds for the dose of graphene, or its derivatives, that may cause cytotoxicity. Ren et al. [188], for example, reported that 50 μg/mL of GO could cause cytotoxicity in fibroblasts, and Lv et al. [189] found that 80 μg/mL of GO could cause both cytotoxicity and decreases in cell viability in cells from the human neuroblastoma cell line SH-SY5Y [188,189]. These findings indicate that the thresholds of the doses at which graphene and its derivatives can exert toxicity vary, possibly depending on the types of cells being tested and the derivatives, do not cause significant levels of toxicity in the host or to cells. Chang et al., for example, evaluated the effects of GO in human lung epithelial cells and found that it did not cause cytotoxicity but did cause oxidative stress in a dose-dependent manner [190]. The use of graphene and its derivatives in the form of films, however, has produced more promising results. Agarwal et al., for example, found that rGO films were not cytotoxic to mouse pheochromocytoma cells, human oligodendroglia cells, or human fetal osteoblasts [191]. Li et al. further found that graphene films with poly-L-lysine were biocompatible with neuronal cells and promoted their growth in vitro [192]. GO films have also been studied by Ruiz et al., who found that the films were biocompatible with mammalian colorectal adenocarcinoma HT-29 cells and improved their proliferation and adhesion [193]. These findings indicate that the forms (e.g., films) of graphene and its derivatives being used can influence the cytotoxicity and efficacy of the materials.

Another limitation of using graphene nanoparticles in biomedical applications involves their shapes, which typically comprise sharp edges. These edges have been found to be damaging to cell membranes as they can penetrate the membranes edge-first, especially when the graphene particles are of a small size [106]. Significant damage to cell membranes can disrupt the normal reparative functions of the cell and cause cytoplasmic contents to leak, which eventually leads to cell death. This issue may be circumvented, however, by utilizing appropriate-sized graphene nanoparticles with larger dimensions. Akhavan et al., for example, found that rGO nanosheets with lateral dimensions of 11 ± 4 nm were more cyto- and genotoxic than their larger counterparts that had lateral dimensions of 3.8 ± 0.4 μm [194]. The smaller nanosheets were also more toxic when administered in large doses of 100 μg/mL, which is consistent with the abovementioned findings regarding the dose-dependent toxicity of graphene. It is important to note, however, that it is easier to remove smaller graphene nanoparticles than larger nanoparticles from organs. Larger GO nanoparticles, for example, have been found to aggregate, which hinders their clearance from the body. Smaller nanoparticles, on the other hand, have been found to be easily removed [195]. Graphene nanoparticles should therefore be of an optimal size that is not too large or small to avoid damaging cells while ensuring the ease of their clearance. 

## 5. Challenges and Future Prospects

Other studies have shown that graphene itself can be non-biodegradable, which can lead to toxicity and hinder effective tissue regeneration [196]. Biodegradability is a key characteristic that allows biomaterials to go from being stable to soluble so that they can be effectively resorbed or excreted from the body. Given the toxicity of accumulated graphene, it is important for graphene nanoparticles to be excreted from the body. Hydrolytic and enzymatic degradation with enzymes such as myeloperoxidase, horseradish peroxidase, and lignin peroxidase have been found to be effective in degrading GO, especially in the presence of low concentrations of hydrogen peroxide [197,198,199,200]. Neutrophils and macrophages have also been reported to cause the degradation of graphene, which indicates the importance of the immune system in mediating the effects of graphene in the body. Girish et al. found that graphene could be phagocytosed by macrophages in vivo and in vitro, which indicates a potential role of the phagocytic immune response in the biodegradation of graphene [201]. Mukherjee et al. further found that GO can be degraded by neutrophils via myeloperoxidase [202]. The degradation products were also assessed for toxicity and were found to be noncytotoxic. Similar results have been reported by Holt et al. [203], who determined that the degradation products of GO are not toxic to cells and that GO-related nanomaterials can be used for long-term TE applications. The question of whether GRMs can be used long-term without harming the body has been another prevalent issue in the field, especially since most studies have only assessed the short-term use of graphene. The findings described by Holt et al. [203] should therefore be further researched, especially in studies assessing the various derivatives of graphene, to fully determine the feasibility of the long-term use of graphene-related biomaterials in TE.

Given the potential of using graphene in biomedical applications, many studies have aimed to determine methods to reduce its toxicity and improve its biocompatibility. Although graphene does not degrade on its own, its carboxylate derivatives can degrade under suitable conditions [204]. Studies have been conducted on the various ways to produce graphene while assessing its related cytotoxic effects. Chemical vapor deposition-produced graphene has been found to increase the generation of ROS, lactate dehydrogenase, and cell death in the brain [205]. It has also been proven that GO has a considerable cytoxic effect on fibroblasts at a concentration of 50 μg/mL. Other studies have shown that human carcinomic alveolar basal epithelial cells (A549) may only become cytotoxic at concentrations of approximately 80 μg/mL, with increased exposure levels eventually leading to apoptosis [188].

Graphene has also been demonstrated to be hazardous in vivo in animal experiments. For example, one study found that after injecting GO into the lungs and liver, GO aggregated in a dose-dependent manner [206]. These negative effects may have been due to the fact that proteins connect to GO non-specifically and that GO is unstable in vivo. The lungs were where GO was mainly aggregated, likely because they are typically the first organs in which graphene-related substances flow after being injected. Other studies have revealed that although the duration of the circulation of GO in the blood was longer than that of other nanomaterials, its absorption in the reticuloendothelial system was minimal. A 14-day study using a GO dose of 1 mg/kg of body weight exhibited its biocompatibility with red blood cells and no pathological changes in the organs examined. Subsequent experiments assessed the capacity of the scaffold to cause thrombosis after being encapsulated with rGO [207]. The adhesion of platelets between the coated and uncoated cellular tissue was not markedly altered. Platelet–leukocyte clumps were not found to adhere, and DNA fragmentation was unchanged. These findings demonstrate the hemocompatibility of rGO, which makes it a potentially promising material for replacing bioprosthetic heart valves [208].

Following functionalization with PEGylation, graphene has been found to act differently in vivo and be far more biocompatible. PEGylated graphene with a diameter between 10 and 50 nm has been found to exhibit low levels of cytotoxicity over the course of 1 month. PEGylated graphene has also been found to accumulate and then be gradually removed over time [209]. The decreased toxicity of PEG-functionalized 125I-labeled nanographene sheets (NGSs) in vitro has further been reported by Yang et al. [180]. Data on the biochemistry, hematocrit levels, and histology of mice were gathered over the course of 3 months following treatment with 20 mg/kg of graphene to assess long-term toxicity. PEGylated NGSs were found to become concentrated in the liver and spleen after being intravenously administered. The NGSs were then excreted and therefore demonstrated good biocompatibility.

Another study examining the impact of GO on medical devices examined the biocompatibility of GO after the implantation of subcutaneous and peritoneal tissue at two oxygenation levels. The findings revealed an inflammatory response that resembled a usual reaction to a foreign body. GO (20 mg/kg) was injected into several tissues with varied C-to-O ratios. Flow cytometry was used to measure the number of inflammatory cells. The macrophage levels were lower in the GO-treated groups than in the control group during the course of a 2-week monitoring period. Notably, larger monocyte numbers and a stronger pro-inflammatory milieu were linked to higher GO oxidation levels. The variables that determine graphene-related toxicity are therefore the dose of graphene used, its size, the treatment period, and the number of layers of graphene. It has also been proposed that the surface characteristics of graphene and its derivatives, such as the functional groups and their chemical structures, are directly connected to the toxicity of the materials. Cytotoxicity may, however, be reduced by crosslinking the derivatives with biocompatible materials or controlling the sizes of the compounds through synthetic methods [210]. 

Graphene-related products can further damage cells and induce oxidative stress. Consequently, while functionalizing graphene and its derivatives, it is crucial to consider their purity. Overall, given that GRMs come in various sizes and shapes and can be produced through various methods, determining and minimizing the toxicity of the materials for clinical usage is essential.

## 6. Conclusions

Overall, studies in the field have produced conflicting results. Many studies have demonstrated the cytotoxicity of graphene nanomaterials in TE applications, whereas others have indicated that they are biocompatible. These inconsistencies may be due to several causes, including the cell types being assessed and their genetic background, experimental techniques and parameters, culture conditions for in vitro experiments, the derivatives and forms of graphene being used, and their physicochemical properties. Further studies are therefore necessary to understand the correlation between these factors and graphene-related cytotoxicity. A larger number of methods to functionalize graphene for use in specific TE applications, such as in neurodevices, targeted drug delivery, implants, DNA sensing, and scaffolds for tissue regeneration, are also needed given the numerous factors that can influence the cytotoxicity and efficacy of graphene-based and -coated materials and devices. Moreover, many studies have assessed the toxicity of graphene and its derivatives in lung or liver cells, but few have studied the cytotoxic effects of graphene on neural cells. Elucidating these issues and further studying the impact of graphene on neural cells will allow for in vivo applications to be more successful, which may eventually lead to human clinical trials taking place and, finally, the commercial and biomedical use of GRMs and devices in nerve TE. 

## Figures and Tables

**Table 1:** Most relevant applications of GRMs in medical field.

Application	GRMs	Comments	Refs.
Tissue engineering	GRMs, in general	GRMS are evaluated in a wide range of applications, in blood vessel, cartilage, muscle or bone tissue engineering. The role of the GRMs is especially related to the enhancement of the mechanical properties.	[60]
Nerve tissue engineering	GRMs, in general: silk fibroin—rGO; PLA-GO, etc.	The presence of the graphenic backbone can assure proper electrical conduction and can assist the differentiation and even the orientation of the neural cells, even the orientation of the nerve tissue constructs.	[127,128,129,130,131,132,133,134,135,136,137]
RNA and DNA analysis	GRMs, in general: rGO-PPy-AuNPs; pure graphene, etc.	Graphene and graphene oxide materials including composites can be used in the analysis of the nucleic acids.	[101,102]
Sensors	Graphene, GO, GQDs, and GOQDs	Graphene oxide and especially graphene oxide at quantum dots level can be specifically accumulate and act as sensors for a wide range of metabolites, peptides, proteins, bacteria, and other medical compounds.	[75,76,77,78]
Drug and gene delivery in teranostics	GRMs, in general	GRMs can be used in teranostics being able to adsorb and release a wide range of antitumoral agents. The sorption and desorption characteristics can be easily tuned according to the nature of the biological active agents and the release needs.	[151,152,153,154,155,156,157,158,159,160]
Drug delivery in the treatment of infections	GRMs, in general	GRMs is used in the treatment of the infections being able to release the antimicrobial agents according to the needs. Additionally, external triggering factors (especially electric fields and infrared radiation) can be used to tune the delivery and thus the antimicrobial activity.	[161,162,163,164,165,166]

## Data Availability

Not applicable.

## Abbreviations

2D, two-dimensional; 3D, three-dimensional; 3DG, 3D printable graphene; 3D-GF, 3D graphene foam; ADSC, adipose stem cell; ApF, antheraea pernyi silk fibroin; AuNP, gold nanoparticle; CBM, carbon-based material; ECM, extracellular matrix; G-NF, graphene nanofiber; GO, graphene oxide; GO-NF, graphene oxide nanofiber; GOQD, graphene oxide quantum dot; GQD, graphene quantum dot; HA, hyaluronic acid; HEK293A, human embryonic kidney cells; HepG2, hepatocellular carcinoma cells; hMSC, human mesenchymal stem cell; HO, hydroxyl radical; HT-29, mammalian colorectal adenocarcinoma cells; HTLV-1, T-lymphotropic virus-1; IPSC, rat-induced pluripotent stem cell; LDI-MS, laser desorption and ionization of mass spectrometry; MAP2, microtubule-associated protein 2; MSC, mesenchymal stem cell; NIH-3T3, fibroblast cell line from a mouse NIH/Swiss embryo; NSC, neural stem cell; PAM, polyacrylamide; PC-12, catecholamine cells; PEF, porcine skin fibroblast; PEG, polyethylene glycol; PLCL, poly(L-lactic acid-co-caprolactone); PPy, polypyrrole; PU, polyurethane; RAW264, monocyte/macrophage-like cells; rGO, reduced graphene oxide; ROS, reactive oxygen species; SAO-2, human osteoblast cells; SC, Schwann cell; SF, silk fibroin; SH-SY5Y, SK-N-SH neuroblastoma cell line; TE, tissue engineering; TUJ1, class III beta tubulin; XPS, X-ray photoelectron spectroscopy.
